# Paradox Resolved? The Strange Case of the Radiation-Resistant Bacteria

**DOI:** 10.1371/journal.pbio.0050108

**Published:** 2007-03-20

**Authors:** Liza Gross

Fifty years ago, scientists experimenting with gamma radiation to sterilize canned foods were surprised to find spoiled meat in cans zapped with what they thought were lethal levels of ionizing radiation (IR). Inside the bulging cans, they discovered a strain of bacteria now called Deinococcus radiodurans. This extremely resilient microbe can endure 100 times the IR levels that kill other bacteria and levels 2,000 times higher than the lethal human dose.

Researchers investigating the nature of radiation toxicity long ago settled on DNA as its principal target. Within this framework, efforts to understand D. radiodurans’s resistance have focused on the mechanisms of DNA repair, with each study revealing seemingly greater levels of efficiency. Surprisingly, this extremophile relies on a set of apparently universal DNA repair proteins, raising an even bigger paradox: DNA repair and synthesis depends on proteins, but these proteins suffer radiation damage, too. And no matter how efficient DNA repair enzymes might be under normal conditions, it’s not clear how they manage to resurrect a radiation-shattered genome if they are also damaged.

Over the past few years, several observations have challenged the DNA-centered view of IR toxicity. For one thing, the D. radiodurans genome, sequenced in 1999, revealed nothing clearly unusual about its DNA repair components. And it appears that bacteria at the opposite ends of resistance sustain about the same amount of DNA damage from a given IR dose, with many bacterial species succumbing to IR doses that cause very little DNA damage. Shewanella oneidensis, for example, cannot survive doses causing less than one double-strand DNA break per genome although it encodes DNA repair systems that appear more complex than those in D. radiodurans, which can weather the 100 double-strand breaks per genome caused by much higher doses just fine.

Might the hypothetical genes identified in the D. radiodurans genome encode proteins with novel repair functions? Or perhaps resistant bacteria can use standard DNA repair equipment in ways other organisms cannot. Or maybe there’s something special in the way the microbe packages its chromosomes.

A 2004 study by Michael Daly et al. found that IR-resistant and IR-sensitive cells had significantly different mineral concentrations, lending support to a role of manganese and iron in recovery. The researchers showed that the most resistant cells contained about 300 times more manganese and three times less iron than the most sensitive cells. In a new study investigating the functional consequences of this disparity, Daly et al. show that high cytosolic manganese and low iron concentrations facilitate resistance by protecting proteins, but not DNA, from IR-induced oxidative damage. Their findings offer a novel perspective on the long-cryptic nature of D. radiodurans resistance, shifting the focus of toxicity and resistance away from DNA damage and repair toward a potent form of protein protection.

**Figure:**
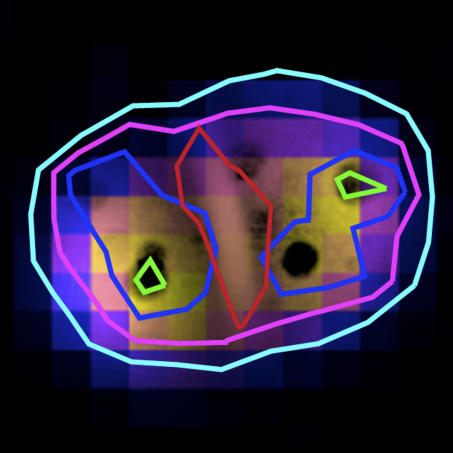
Image overlay of transmission electron microscopy, light microscopy, and X-ray fluorescence microprobe analyses of D. radiodurans. Average abundance of manganese (blue, green, and pink) and iron (red) are shown within a single D. radiodurans diplococcus.

Exposing cells to IR generates a range of potentially harmful molecules called reactive oxygen species (ROS). When ROS accumulate faster than cellular scavengers can neutralize them, they cause oxidative stress and can kill cells. Hydroxyl radicals, one of the primary ROS products of irradiated water (the major component of cells), are particularly toxic to DNA, and can generate other ROS, including hydrogen peroxide and superoxide (a simple peroxyl radical).

High intracellular concentrations of manganese ions are known to alleviate oxidative stress in several bacterial species; these ions can interact with different ROS depending on their oxidation state and their binding with different molecules. Daly et al. reasoned manganese might affect ROS generation during irradiation. They first tested manganese’s ability to scavenge hydroxyl and superoxide radicals to determine whether its activity protects DNA or proteins. Whereas hydroxyl radicals target both DNA and proteins, superoxide radicals selectively damage proteins. The researchers irradiated DNA and a DNA-modifying enzyme and found that, although manganese ions failed to protect DNA from hydroxyl radicals generated during irradiation, the ions did prevent enzyme damage and preserved enzyme activity.

To understand the nature of manganese protection in cells, the researchers then irradiated IR-sensitive and IR-resistant bacteria and compared their levels of oxidative protein damage. The sensitive cells with the lowest manganese to iron concentration ratios, they found, sustained high levels of protein oxidation; the resistant cells with the highest ratios had no detectable protein oxidation. They showed that proteins purified from D. radiodurans are not inherently oxidation-resistant, and when cells were depleted of manganese, cells were rendered sensitive to IR and protein oxidation. This suggests that the microbe actively offsets the effects of IR by protecting proteins using manganese, specifically with divalent manganese (Mn(II)) ions.

**Figure:**
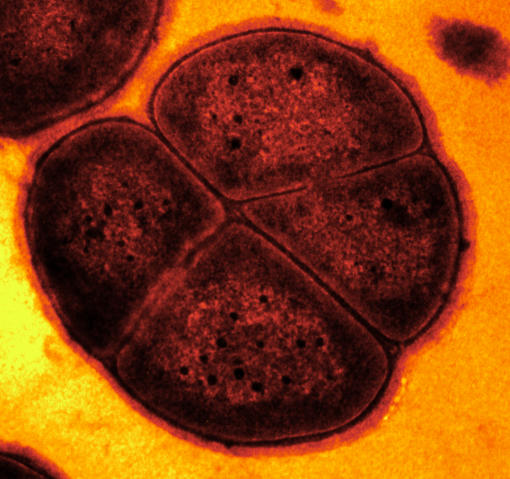
Electron micrograph of a cross-section of a D. radiodurans tetracoccus (cluster of four cells).

Resistant bacteria, the researchers suspected, might use Mn(II) to transform superoxide radicals, which can’t easily cross the cell membrane, into hydrogen peroxide, which can. And that’s what they found: irradiated D. radiodurans and a second resistant bacteria with high manganese concentrations (Lactobacillus plantarum) released hydrogen peroxide (likely as a product of the “redox” reactions that neutralize superoxide radicals), while sensitive and non-irradiated resistant bacteria did not. The researchers went on to show that the resistance of normal D. radiodurans can be controlled externally by inhibiting manganese redox recycling.

In the context of previous studies, these results suggest that D. radiodurans relies not on a highly specialized DNA repair machinery, but on a detoxifying mechanism associated with the microbe’s unusual intracellular environment. Most organisms contain near-millimolar concentrations of iron, which under IR will contribute to the formation of hydroxyl radicals and superoxide radicals. In resistant bacteria, millimolar Mn(II) concentrations appear to protect proteins from oxidative damage by eliminating superoxide and its derivatives. This oxidative protection may in turn shield proteins involved in DNA repair, and subsequently allow them to quickly heal DNA lesions, which in sensitive bacteria turn lethal because their repair proteins are ravaged by radiation.

This new model of radiation toxicity opens up novel avenues for radioprotection in diverse settings. Individuals exposed to chronic or acute doses of radiation could potentially benefit from treatments that deliver purified D. radiodurans Mn complexes into their cells. Similarly, the toxic effects of radiation therapy in cancer patients might be ameliorated by antioxidant drugs based on such a protection paradigm. And given that many bacteria, such as S. oneidensis, with favorable bioremediation functions are extremely sensitive to radiation, the new insight on how D. radiodurans survives radiation might prove useful in efforts to contain the toxic runoff from the immense radioactive- and heavy-metal-contaminated waste sites left over from the Cold War.

